# *Brucella melitensis* in Asian Badgers, Northwestern China

**DOI:** 10.3201/eid2604.190833

**Published:** 2020-04

**Authors:** Xiafei Liu, Meihua Yang, Shengnan Song, Gang Liu, Shanshan Zhao, Guangyuan Liu, Sándor Hornok, Yuanzhi Wang, Hai Jiang

**Affiliations:** Shihezi University, Shihezi, China (X. Liu, M. Yang, S. Song, G. Liu, S. Zhao, Y. Wang);; Lanzhou Veterinary Research Institute, Lanzhou, China (G. Liu);; University of Veterinary Medicine, Budapest, Hungary (S. Hornok);; Chinese Center for Disease Control and Prevention, Beijing, China (H. Jiang)

**Keywords:** Brucella melitensis, bacteria, Asian badger, Meles leucurus, zoonoses, China

## Abstract

We isolated *Brucella melitensis* biovar 3 from the spleen of an Asian badger (*Meles leucurus*) in Nilka County, northwestern China. Our investigation showed that this isolate had a common multilocus variable-number tandem-repeat analysis 16 genotype, similar to bacterial isolates from local aborted sheep fetuses.

Brucellosis can be transmitted between domestic animals and wildlife (*1*). *Brucella melitensis* has been isolated from wildlife, such as chamois (*Rupicapra rupicapra*) (*2*), Alpine ibex (*Capra ibex*) (*3*), and Iberian wild goat (*Capra pyrenaica*) (*4*). Badgers are major predators in forests and consume a broad spectrum of food items, including small terrestrial vertebrates and their cadavers (*5*), which might result in contact with pathogens from tissues of these vertebrates. We report an Asian badger (*Meles leucurus*) in China naturally infected with *B. melitensis* biotype 3.

This study was approved by the Animal Ethics Committee of Shihezi University (approval no. AECSU2017–04). In 2017, a total of 7 illegally hunted and dying badgers in Nilka County, northwestern China, were confiscated by the local government.

We identified the animals as Asian badgers by using a PCR targeting the 16S rDNA gene (GenBank accession no. MH155253). We collected different organs or tissues, including heart, liver, spleen, lung, kidney, small intestine, large intestine, and blood, from all badgers. We separated serum from blood samples by centrifugation at 1,000 × *g* for 15 min and tested serum by using the rose bengal test (RBT) and serum agglutination test (SAT) (*6*). To detect *Brucella* antigens, we used immunohistochemical staining of liver and spleen tissue sections by pipetting mouse anti–*Brucella melitensis* IgG diluted 1:100 in 30% bovine serum albumin/phosphate-buffered saline onto each section. For comparison, we collected samples from 14 aborted sheep fetuses from Nilka County.

We extracted genomic DNA from all samples by using a commercial kit (Blood and Cell and Tissue Kit; BioTeke, http://www.bioteke.com). We used the partial *omp*22 gene (238 bp) encoding 22-kD outer membrane protein to identify the *Brucella* genus and the *IS*711 gene to identify *Brucella* species. We used PCRs that have been described (*7*). We used *Brucella* reference strains (*B. melitensis* 16M and *B. abortus* 2308) as positive controls and double-distilled water as a negative control.

We homogenized spleen samples of badgers and the 14 aborted sheep fetuses and inoculated these homogenates onto individual *Brucella* agar plates, which we then incubated at 37°C in an atmosphere of 5% CO_2_ for 5 days. We tested putative *Brucella* colonies by using H_2_S production, dye inhibition, agglutination by monospecific serum, and sensitivity to bacteriophages (Appendix Table). We analyzed colonies by using a multilocus variable-number tandem-repeat analysis (MLVA) typing assay (*8*).

Only serum from badger no. 2 was positive for smooth *Brucella* antigen by RBT and SAT; the specific antibody titer was 1:160 (≈125 IU/mL). We successfully amplified 2 genetic markers (regions of the *omp*22 and *IS*711 genes) from blood, heart, liver, spleen, lung, kidney, small intestine, and large intestine from badger no. 2 but not from samples of other badgers. In addition, we isolated *B. melitensis* biotype 3 from badger no. 2 and 5 aborted sheep fetuses according to phenotypic identification (Appendix Table). MLVA-16 typing indicated that the isolates from badger no. 2 and aborted sheep fetuses had a common MLVA-16 type (1-5-3-13-2-2-3-2-4-40-8-8-4-3-7-7). In addition, immunohistochemical staining with a brown chromogen (diaminobenzidine) identified *Brucella* antigens in liver and spleen of badger no. 2 (Figure).

**Figure:**
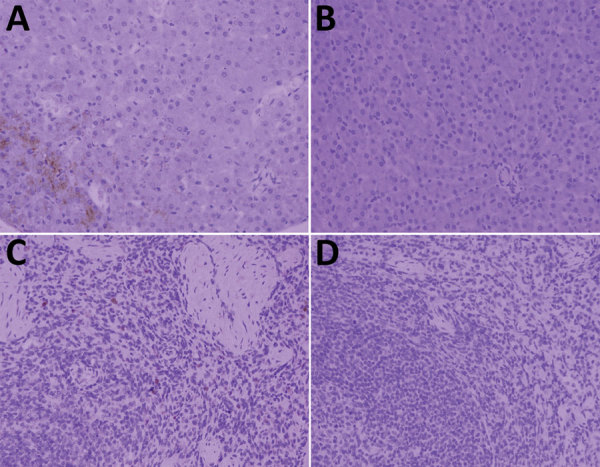
*Brucella* antigen in liver and spleen from Asian badgers infected with *Brucella melitensis*, northwestern China. A) *Brucella* antigen in liver of Asian badger no. 2; B) liver of an uninfected Asian badger; C) *Brucella* antigen in spleen of Asian badger no. 2; D) spleen of badger without *Brucella* antigen. Diaminobenzidine staining; original magnification ×400.

*B. melitensis* is isolated mainly from goats and sheep, in which it causes fetal abortion (*1*). The Asian badger is a semihibernating, burrowing animal species that has not been reported to harbor this pathogen. In a previous study, Li and Hu reported that 0.30% (12/4,015) of sheep in Nilka County, China, were serologically positive for smooth *Brucella* antigen by RBT and 9.75% (145/1,485) were serologically positive for smooth *Brucella* antigen by SAT (*9*). The habitats of Asian badgers and the grazing areas of sheep and goats partially overlap, which can be most likely explained by observations of shepherds that Asian badgers eat aborted fetuses or their placentas during lambing season in winter. In this study, *B. melitensis* biovar 3 isolates, designated as XJ1802 and XJ1804 strains, were found in aborted sheep fetuses and an Asian badger. MLVA-16 typing indicated that they shared a common MLVA-16 type (Appendix Figure). This finding suggests that the Asian badger is a *Brucella* spillover host that becomes infected from sheep that act as a reservoir host.

Another study reported that coyotes were infected probably through ingestion of aborted fetuses and placentas in enzootic brucellosis areas (*10*). In our study, we detected *Brucella* DNA from blood, heart, liver, spleen, lung, kidney, small intestine, and large bowel of badger no. 2 and identified *B. melitensis* biovar 3 from spleen tissue. This finding suggests that pathologic changes in multiple organs or tissues caused by *B. melitensis* might occur.

In the future, it will be essential to evaluate the clinical status of Asian badgers naturally infected with *B. melitensis*. In addition, more extensive surveillance is necessary to expand our knowledge on the epidemiologic interface between wildlife and domestic animals in the context of *Brucella* infections.

Appendix Additional information on *Brucella melitensis* in Asian badgers, northwestern China.
