# Supplementary material for: Brucella melitensis in Asian Badgers, Northwestern China
Source: Emerg Infect Dis. 2020 Apr;26(4):804–6. doi: 10.3201/eid2604.190833 (PMC7101117; doi:10.3201/eid2604.190833)
Supplement: Appendix — Additional information on Brucella melitensis in Asian badgers, northwestern China. [file 19-0833-Techapp-s1.pdf]

Article DOI: https://doi.org/10.3201/eid2604.190833

Brucella melitensis in Asian Badgers, Northwestern China

Appendix

Appendix Table. Characteristics of *Brucella* isolates from Asian badger and other sources, northwestern China*

Characteristic	*Brucella* isolate in spleen of Asian badger	*Brucella* isolates in aborted sheep fetuses (n = 5)	International reference strains		
			16M	544A	1330s
CO_2 requirement	–	–	–	–	–
H_2S produced†	–	–	+	++	+++
Dye inhibition‡					
Thionin	+	+	+	–	+
Basic fuchsin	+	+	+	+	–
Lysis by bacteriophage at RTD					
Tb RTD	–	–	–	+	–
Tb 10^4 RTD	–	–	–	+	+
Iz	–	–	–	–	–
BK2	+	+	+	+	+
Agglutination of monospecific serum					
A	+	+	–	+	+
M	+	+	+	–	–
R	–	–	–	–	–
Species	*B. melitensis*	*B. melitensis*	*B. melitensis*	*B. abortus*	*B. suis*
Biotype	3	3	1	1	1

*RTD, routine test dilution; –, negative; +, positive.

†+, weakly positive; ++, positive; +++, strongly positive.

‡Dye concentration in *Brucella* agar medium is 20 μg/mL (1:50,000 dilution).

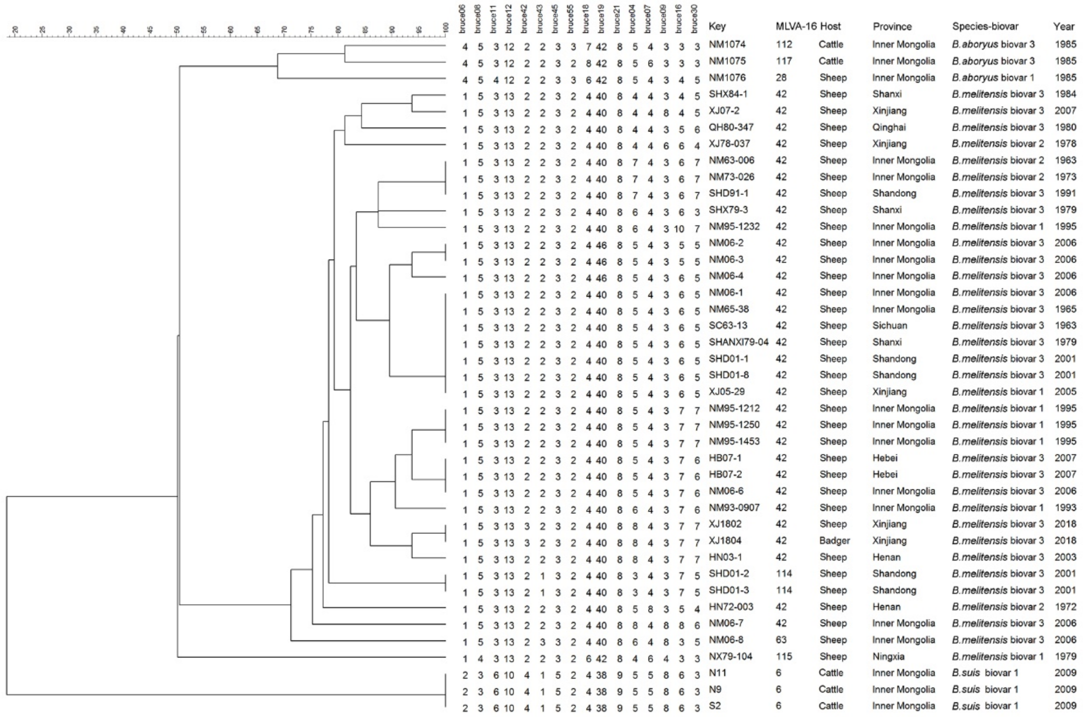

Appendix Figure. Dendrogram based on the MLVA-16 genotyping assay showing relationship between *Brucella* isolates from an Asian badger and aborted sheep fetuses and other bacteria, northwestern China. Shown are serial numbers for *Brucella* isolates; MLVA-16 genotype and host from which the bacteria was isolated; province; Species-biovar (biotyping result corresponding to each isolate); and year of isolation. Scale bar indicates percent relatedness. MLVA, multiple locus variable number tandem repeat analysis typing assay
